# Fine-scale genetic differentiation of a temperate herb: relevance of local environments and demographic change

**DOI:** 10.1093/aobpla/plu070

**Published:** 2014-11-10

**Authors:** Yasuhiro Sato, Hiroshi Kudoh

**Affiliations:** Center for Ecological Research, Kyoto University, Hirano 2–509-3, Otsu, Shiga 520-2113, Japan

**Keywords:** *Arabidopsis halleri* subsp. *gemmifera*, Brassicaceae, genetic structure, isolation-by-adaptation, isolation-by-distance, Japan.

## Abstract

The genetic structure of a plant species is shaped by environmental adaptation and demographic factors, but their relative contributions are still unknown. To examine the environment- or geography-related differentiation, we quantified genetic variation among 41 populations of a temperate herb, *Arabidopsis halleri* subsp. *gemmifera* (Brassicaceae). We analysed 19 microsatellite loci, which showed a significant population differentiation and a moderate within-population genetic diversity (global *G*_st_ = 0.42 and *H*_s_ = 0.19). Our structure analysis and phylogenetic network did not detect more than two genetic groups across the Japanese mainland but found fine-scale genetic differentiations and admixed patterns around the central area. Across the Japanese mainland, we found significant evidence for isolation-by-distance but not for isolation-by-environments. However, at least within the central area, the magnitude of genetic differentiation tended to increase with microhabitat dissimilarity under light conditions and water availability. Furthermore, most populations have been estimated to experience a recent decline in the effective population size, indicating a possibility of bottleneck effects on the pattern of genetic variation. These findings highlight a potential influence of the microhabitat conditions and demographic changes on the local-scale genetic differentiation among natural plant populations.

## Abstract (precis)

To examine determinants of geographical patterns in population differentiation, we quantified genetic variation of a temperate herb, *Arabidopsis halleri* subsp. *gemmifera*, across the Japanese mainland. We found significant evidence for isolation-by-distance but not for isolation-by-environments. However, at least within the central area, the magnitude of genetic differentiation tended to increase with microhabitat dissimilarity in light conditions and water availability. Furthermore, most populations have been estimated to experience a recent decline in the effective population size. These findings highlight a potential influence of the microhabitat conditions and demographic processes on the local-scale genetic differentiation among natural plant populations.

## Introduction

Current patterns of genetic structure within a single plant species are shaped by demographic (e.g. genetic drift or gene flow) and ecological (e.g. species' life-history traits or adaptation to local environments) factors (Sharbel *et al.* 2000; Platt *et al.* 2010; Willi and Määttänen 2011). At a regional scale where current migrations are probably limited (e.g. on a several hundred-kilometre scale), many phylogeographical studies have revealed historical influences on the genetic structure of natural populations (e.g. Fujii and Senni 2006; Garrido *et al.* 2012; Meeus *et al.* 2012). For example, impacts of glacial periods upon temperate flora were prominent in the regional patterns of genetic differentiation, since they have been relevant to latitudinal or altitudinal migrations, population extinction, range expansion and fragmentation (Hewitt 2000). On the other hand, at a local scale where ongoing migrations and drifts are possible, landscape genetic studies have reported that habitat fragmentations and life-history traits of each plant species can be determinants of within-population genetic diversity and genetic differentiation among proximate populations, because they are involved in limited seed or pollen dispersal and genetic drifts (e.g. Kudoh and Whigham 1997; Vekemans and Hardy 2004; Bomblies *et al.* 2010).

In addition to ample evidence on the genetic structure of plant populations from phylogeographical and landscape studies, there has also been increasing evidence that local environment and geographic scale exert mutually non-exclusive influences on the pattern of genetic structure (Nosil *et al.* 2009; Sobel *et al.* 2010; Lee and Mitchell-Olds 2011). For example, environmental factors may contribute to genetic differentiation by mediating local adaptation that can reduce the fitness of immigrants or promote reproductive isolation. These conceptual frameworks, which are termed ‘ecological speciation’ (Sobel *et al.* 2010) or ‘isolation-by-adaptation’ (Nosil *et al.* 2009), have been recognized as important. Thus, for a comprehensive understanding of an evolutionary trajectory of plant populations, it is necessary to consider the interaction between ecological and demographic processes.

The Japanese archipelago consists of four main islands extending over 2000 km in the southwest–northeast direction and encompassing a wide range of climatic zones, including many floral or vegetation types from warm-temperate, evergreen and broadleaf forests to temperate, deciduous and sub-boreal coniferous forests (Numata 1974). No major glaciers were present in Japan during the last glacial period, but the temperature and precipitation were significantly lower than that at present (Tsukada 1988). Reconstruction of past vegetation from palaeoecological (Takahara *et al.* 2000) and phylogeographical data (Fujii and Senni 2006) indicates that the main vegetation zones were displaced towards the south and lower altitudes (see Dobson 1994). In the temperate zone of Japan in particular, the genetic structure of deciduous broadleaved tree species showed a regional genetic differentiation between the coastal areas of the sea of Japan and the Pacific Ocean (Hiraoka and Tomaru 2009*a*; Iwasaki *et al.* 2012). These regional-scale investigations postulate a phylogeographically unique scenario of recent range expansions from the central region along coastal areas of the Japanese mainland. Accumulated phylogeographical evidence also suggests that historical climate changes have shifted the distribution range, and consequently altered the genetic diversity of Japanese vegetation (e.g. Sakaguchi *et al.* 2010, 2011). However, local-scale analyses are still limited regarding the pattern of genetic differentiation and its association with local environments.

*Arabidopsis halleri* subsp. *gemmifera* (syn. *Arabis gemmifera*) is distributed throughout Japan and the Russian Far East (Hoffmann 2005), where regional scales for this subspecies can be considered to be several hundred kilometres or more. *Arabidopsis halleri* subsp. *gemmifera* can reproduce clonally: after flowering, plants produce new rosettes on the main and lateral meristems of flowering stems (Aikawa *et al.* 2010), and these rosettes often establish as clonal offspring once they have rooted and attached themselves to the ground. The flowers are self-incompatible and produce fruits through cross-pollination (Tsuchimatsu *et al.* 2012). The pollinators are small solitary bees and flower flies, and the seeds have no specific structure for long-distance dispersal. This subspecies is often found in somewhat isolated habitats near open gravel sites along valleys or in gaps within vegetation (Ihara 1976). The sporadic distribution, together with the lack of long-distance dispersal, may therefore enhance the genetic structure formed at a local scale (<10 to a few hundred kilometres). Furthermore, *A. halleri* subsp. *gemmifera* often occurs near human-disturbed habitats such as forest pathways and abandoned mines, whereby artificial dispersals may homogenize the local genetic structure.

In this study, we examined genetic differentiation among natural populations of *A. halleri* subsp. *gemmifera.* In particular, we conducted a fine-scale sampling around the Kinki area, because private genetic groups or admixed populations have often been reported for its coastal regions in temperate plants of Japan (Hiraoka and Tomaru 2009*b*; Iwasaki *et al.* 2010). We used cross-species microsatellite markers developed for a close relative, *A. thaliana*, to detect genetic structures at a fine resolution. Microsatellite loci are known to be highly polymorphic (e.g. Emanuelli *et al.* 2013) and consequently provide us with suitable levels of variation for quantifying genetic differentiation at a fine scale. Three specific questions were addressed in this study. Is there any distinct genetic structure among *A. halleri* subsp. *gemmifera* populations? Is the magnitude of genetic differentiation associated with environmental gradients and/or geographical distance? Are demographic processes involved in the genetic differentiation? To answer these questions, we initially generated a Bayesian clustering and phylogenetic network to determine the patterns of genetic differentiation. Secondly, we analysed the isolation-by-distance or -by-environmental differences. Finally, to estimate the demographic processes of each population, declines/expansions of effective population sizes were tested using a coalescence-based method.

## Methods

### Study sites, plant materials and DNA extraction

**Figure 1.**
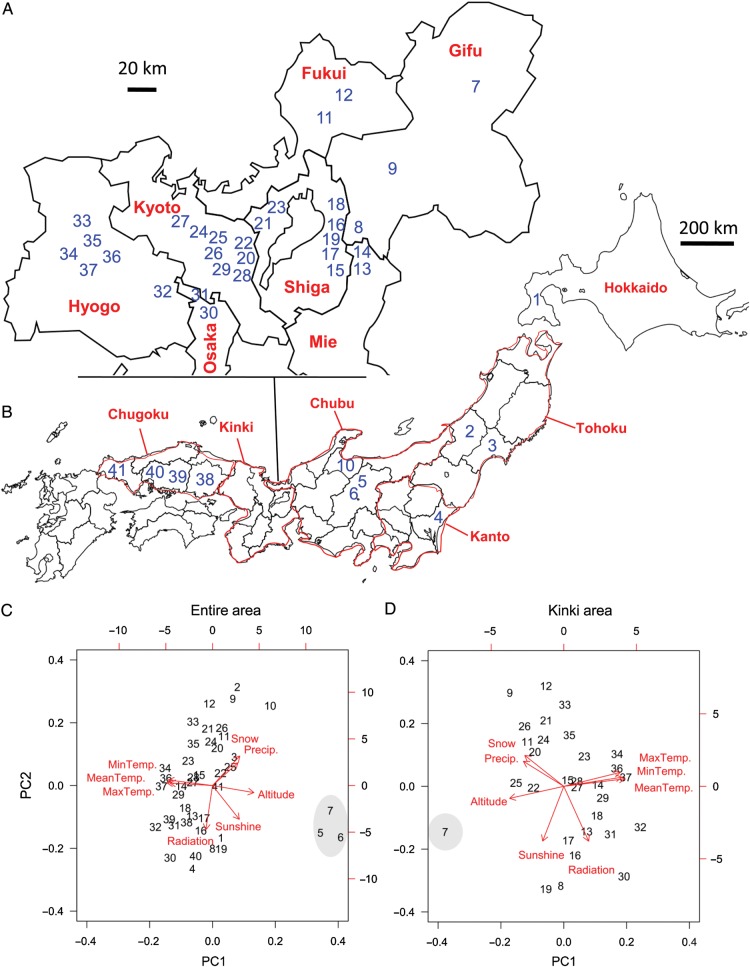
Map of sampling sites (A and B) and two principal components (PCs) summarizing eight environmental variables (C and D) for 41 populations of *A. halleri* subsp. *gemmifera*. The site IDs (coloured blue) are mapped on the sampling location. (A) focuses on the Kinki area whereas (B) presents the entire study area. The eight environmental variables include air temperature (°C, daily mean, maximum and minimum), annual precipitation (mm), radiation (h), sunshine (MJ/m^2^), maximum snow cover (cm) and altitude (m). PC1 and PC2 are also shown for the entire study area (C) and the Kinki area (D). Red arrows represent the contributions of each environmental variable to the PCs. Numbers correspond to the site IDs. Note that high-altitude populations (shaded IDs) show outlier values beyond the plot area.

We studied 41 populations in Japan; i.e. 30 sites located within the Kinki area and its surroundings (referred to hereafter as the Kinki area) and 11 sites far from the Kinki area (hereafter, as outside the Kinki area) (Table 1; Fig. 1A and B). *Arabidopsis halleri* subsp. *gemmifera* often inhabits habitats along valleys or forest margins, where the size of study sites roughly ranged from hundreds to thousands of metres in transect length **[****see Supporting Information****]**. We sampled fresh, young leaves from 4 to 14 individuals for each sampling site (Table 1). Sampled plants were at least 1 m apart from each other to minimize the possibility of multiple sampling from single clonal lineages. Total DNA was extracted using a DNeasy Plant Mini kit (Qiagen), CTAB (Doyle and Doyle 1987) or miniscale DNA extraction method (Goto 2005) and stored at −20 °C until use.

**Table 1.** List of 41 populations of *A. halleri* subsp. *gemmifera* and their genetic characteristics. Site ID, locality name, latitude, longitude and altitude are shown for each sampling site. The number of plants sampled per site (*n*), the number of polymorphic loci (#Poly. loci), the number of private alleles per site (#PA), AR, observed heterozygosity, within-population genetic diversity (*H*_o_ and *H*_s_) and inbreeding coefficient (*G*_is_) are shown based on the results from 19 microsatellite loci. Bold values of *G*_is_ indicate <5 % significant deviation from the Hardy–Weinberg equilibrium values, in which positive and negative values indicate deficit and excess of heterozygotes, respectively. Demographic estimates by the Msvar programme (current and ancient effective population size, *N*_0_ and *N*_1_, and time since the change of the population size, *T*_a_) are also given. Mean and 95 % highest posterior density intervals (within square brackets) are presented for the Msvar estimates. Scale bars (---) indicate a study site not analysed due to its limited sample size, and NA means no information available.

Site ID	Name	Latitude	Longitude	Alt. (m)	*n*	#Poly. loci	#PA	AR	*H*_o_	*H*_s_	*G*_is_	log_10_(*N*_0_)	log_10_(*N*_1_)	log_10_(*T*_a_)
1	Hakodate, Hokkaido	41°47′N	140°49′E	10	10	4	0	1.23	0.10	0.08	**−0.25**	1.7 [0.2–2.7]	5.1 [3.5–7.0]	4.5 [2.6–6.7]
2	Mazawa, Yamagata	38°26′N	140°08′E	210	10	10	0	1.45	0.18	0.19	**0.07**	2.1 [0.8–3.1]	5.0 [3.7–6.2]	3.9 [2.4–5.4]
3	Okunikkawa, Miyagi	38°20′N	140°36′E	320	4	10	2	1.63	0.25	0.26	0.05	---	---	---
4	Fukuroda, Ibaraki	36°46′N	140°24′E	120	6	8	0	1.34	0.16	0.14	−0.10	---	---	---
5	Kaida, Nagano	35°59′N	137°36′E	1270	10	11	1	1.66	0.28	0.28	0.01	2.2 [1.1–3.1]	5.1 [3.8–6.3]	4.0 [2.6–5.2]
6	Kamikochi, Nagano	36°15′N	137°39′E	1510	6	12	0	1.53	0.15	0.20	**0.23**	---	---	---
7	Hirayu, Gifu	36°11′N	137°33′E	1260	8	9	0	1.42	0.14	0.16	0.12	2.2 [1.2–3.1]	4.6 [3.5–5.7]	4.0 [2.7–5.3]
8	Sofudani, Gifu	35°19′N	136°27′E	190	8	11	0	1.68	0.21	0.27	**0.24**	2.8 [1.8–3.6]	5.1 [2.8–7.9]	5.6 [2.3–9.9]
9	Midoridani, Gifu	35°37′N	136°36′E	260	12	9	0	1.47	0.12	0.17	**0.26**	2.6 [1.9–3.3]	5.7 [3.9–7.5]	5.5 [3.9–7.3]
10	Funato, Toyama	36°28′N	137°14′E	225	6	9	1	1.58	0.14	0.21	**0.34**	---	---	---
11	Takefu, Fukui	35°52′N	136°15′E	120	10	10	0	1.58	0.14	0.21	**0.33**	2.4 [1.5–3.3]	5.3 [4.2–6.4]	4.6 [3.2–6.0]
12	Katsuyama, Fukui	36°03′N	136°29′E	150	10	8	0	1.38	0.12	0.16	**0.26**	2.2 [1.4–3.1]	5.2 [3.8–6.5]	4.6 [3.0–6.1]
13	Fujiwara-Mikuni, Mie	35°13′N	136°27′E	240	9	12	1	1.95	0.33	0.34	0.04	2.6 [1.6–3.5]	4.6 [3.4–5.8]	4.1 [2.5–5.7]
14	Fujiwara-Sakamoto, Mie	35°11′N	136°28′E	250	6	9	1	1.67	0.17	0.23	**0.28**	---	---	---
15	Kiwada, Shiga	35°06′N	136°22′E	310	10	11	0	1.50	0.13	0.19	**0.33**	2.6 [1.9–3.3]	5.2 [4.0–6.5]	4.5 [3.4–5.6]
16	Ryozen-Niu, Shiga	35°18′N	136°22′E	210	6	9	3	1.65	0.18	0.23	**0.21**	---	---	---
17	Ojigahata, Shiga	35°13′N	136°23′E	310	10	11	0	1.66	0.22	0.25	**0.12**	2.0 [1.0–2.9]	4.9 [4.1–5.7]	3.5 [2.5–4.5]
18	Ibuki, Shiga	35°24′N	136°23′E	350	7	10	0	1.56	0.20	0.23	0.15	---	---	---
19	Gongendani, Shiga	35°15′N	136°22′E	380	6	11	0	2.02	0.31	0.36	0.14	---	---	---
20	Asibidani, Shiga	35°13′N	135°51′E	410	9	10	0	1.59	0.10	0.22	**0.55**	2.6 [1.8–3.3]	5.4 [4.2–6.6]	4.6 [3.5–5.6]
21	Umenoki, Shiga	35°16′N	135°52′E	430	10	6	0	1.33	0.01	0.14	**0.92**	2.2 [1.4–3.0]	6.1 [4.5–7.7]	4.9 [3.6–6.3]
22	Katsuragawa-Sakashita, Shiga	35°11′N	135°51′E	480	6	10	0	1.42	0.14	0.18	**0.22**	---	---	---
23	Kutsuki, Shiga	35°22′N	135°55′E	180	14	10	0	1.47	0.18	0.20	0.10	2.3 [1.4–3.1]	4.9 [3.5–6.1]	4.4 [2.7–5.9]
24	Hanase-Yamasu, Kyoto	35°13′N	135°47′E	410	10	6	0	1.18	0.09	0.08	−0.09	2.0 [1.1–2.8]	5.4 [4.3–6.6]	4.4 [3.3–5.7]
25	Hanase-Bessho, Kyoto	35°11′N	135°47′E	500	10	9	0	1.41	0.15	0.16	0.05	2.4 [1.7–3.1]	5.4 [4.2–6.5]	4.9 [3.7–6.2]
26	Miyama, Kyoto	35°18′N	135°42′E	350	10	9	0	1.40	0.16	0.19	0.15	2.2 [1.4–3.0]	5.3 [4.2–6.5]	4.2 [3.2–5.2]
27	Kurama, Kyoto	35°08′N	135°47′E	420	10	4	0	1.12	0.06	0.06	−0.07	2.1 [1.2–2.8]	5.8 [3.9–7.6]	5.2 [3.7–6.9]
28	Ohara, Kyoto	35°10′N	135°51′E	330	10	10	0	1.62	0.13	0.23	**0.43**	2.6 [1.8–3.3]	5.2 [4.0–6.2]	4.7 [3.5–5.9]
29	Shizuhara, Kyoto	35°07′N	135°48′E	230	10	7	0	1.45	0.10	0.16	**0.38**	2.1 [1.0–3.2]	5.1 [3.6–6.7]	4.3 [2.7–5.9]
30	Minoo, Osaka	34°51′N	135°28′E	160	10	10	0	1.57	0.16	0.20	**0.22**	2.5 [1.6–3.4]	4.7 [2.9–6.2]	4.3 [2.7–5.9]
31	Myoken, Osaka	34°55′N	135°27′E	230	10	10	0	1.67	0.24	0.24	−0.01	2.4 [1.5–3.3]	4.9 [3.8–6.0]	4.2 [2.8–5.5]
32	Tada, Hyoto	34°54′N	135°21′E	140	10	10	0	1.57	0.19	0.23	**0.15**	2.4 [1.4–3.2]	5.1 [3.7–6.3]	4.3 [2.9–5.7]
33	Mikohata, Hyogo	35°15′N	134°43′E	320	10	9	1	1.47	0.16	0.18	**0.08**	1.8 [0.7–2.8]	4.9 [4.0–5.8]	3.6 [2.4–4.7]
34	Takacho-Tada, Hyogo	35°06′N	134°53′E	190	10	8	0	1.35	0.16	0.15	−0.04	2.5 [1.8–3.2]	5.9 [2.1–9.8]	8.2 [4.6–12]
35	Ikuno, Hyogo	35°10′N	134°49′E	360	10	10	0	1.55	0.22	0.23	**0.04**	2.5 [1.5–3.4]	5.4 [3.7–6.9]	4.7 [2.9–6.2]
36	Omoide-gawa, Hyogo	35°06′N	134°56′E	200	9	9	0	1.35	0.15	0.16	0.05	2.5 [1.8–3.2]	6.0 [4.1–7.8]	5.2 [3.8–6.9]
37	Monzen, Hyogo	35°05′N	134°54′E	160	10	10	0	1.40	0.16	0.18	0.07	2.6 [1.9–3.3]	5.8 [3.8–7.5]	5.3 [3.6–7.2]
38	Fukiya, Okayama	34°52′N	133°27′E	280	10	5	0	1.22	0.05	0.08	**0.35**	2.0 [1.0–2.8]	5.3 [3.8–6.9]	4.5 [3.2–6.0]
39	Uga, Hiroshima	34°33′N	132°23′E	80	6	4	0	1.16	0.12	0.08	**−0.59**	---	---	---
40	Enmeikyo, Hiroshima	34°42′N	133°22′E	280	7	0	0	1.00	0.00	0.00	NA	---	---	---
41	Tokusa, Yamaguchi	34°26′N	131°41′E	550	10	5	0	1.23	0.10	0.10	0.09	2.2 [1.5–3.0]	6.0 [2.2–10]	8.2 [4.7–12]

### Microsatellite analysis

A preliminary investigation used 40 primers previously reported for *Arabidopsis* species (Bell and Ecker 1994; Loridon *et al.* 1998; Clauss *et al.* 2002; Symonds and Lloyd 2003; Mable and Adam 2007; Llaurens *et al.* 2008). We selected 19 markers that were successfully amplified, regardless of the amount of polymorphism. Three primer set combinations (Loci 8, 4 and 7) were prepared for multiplex PCR with fluorescent dyes (Table 2) so that similar fragment sizes could be distinguished by different dyes. For each sample, we determined the genotypes of 19 loci based on the distinct electrophoretic mobility of amplified fragments <50 bp.

**Table 2.** Primer information and basic population genetic statistics for microsatellite loci used in this study. Multiplex combinations (comb. 1–3), four types of inflorescent dye, annealing temperature (°C), size range of fragments detected in this study and the number of alleles are shown for each locus. Observed heterozygosity, within-population and total genetic diversity (*H*_o_, *H*_s_ and *H*_t_, respectively); inbreeding coefficients (*G*_is_: bold values indicate <5 % significant deviations from the Hardy–Weinberg equilibrium); and population differentiation (*G*_st_: bold values indicate <5 % significant deviations from no differentiation). See also references for sequence information. Scale bars (---) indicate that the summary for overall loci are not applicable to these information.

Primer	Dye	Tm	Comb	Range (bp)	#Allele	Reference	Heterozygosity and *G*-statistics				
							*H*_o_	*H*_s_	*H*_t_	*G*_is_	*G*_st_
ATH	FAM	53	1	152–158	4	Llaurens *et al.* (2008)	0.10	0.13	0.30	**0.26**	**0.55**
ELF3	FAM	53	1	267–300	10	Llaurens *et al.* (2008)	0.44	0.53	0.79	**0.18**	**0.33**
LYR133	VIC	53	1	130–150	6	Llaurens *et al.* (2008)	0.09	0.12	0.21	**0.24**	**0.42**
ICE12	VIC	53	1	226–236	5	Clauss *et al.* (2002)	0.19	0.22	0.30	**0.16**	**0.25**
ICE5	PET	53	1	173–177	2	Clauss *et al.* (2002)	0.02	0.02	0.03	**0.02**	**0.48**
nga112	NED	53	1	173–205	12	Clauss *et al.* (2002)	0.39	0.48	0.82	**0.19**	**0.42**
ICE8	VIC	53	1	50–60	2	Clauss *et al.* (2002)	0.00	0.01	0.01	**1.00**	−0.02
ICE14	FAM	53	1	219–237	6	Clauss *et al.* (2002)	0.16	0.16	0.31	**0.06**	**0.46**
nga361	NED	58	2	124–134	6	Llaurens *et al.* (2008)	0.37	0.42	0.58	**0.12**	**0.29**
nga1145	VIC	58	2	225–237	6	Symonds and Lloyd (2003)	0.25	0.31	0.58	**0.20**	**0.47**
AthCTR1A	VIC	58	2	150–156	4	Bell and Ecker (1994)	0.12	0.18	0.36	**0.33**	**0.51**
MHJ24	FAM	58	2	129–135	3	Clauss *et al.* (2002)	0.02	0.04	0.04	**0.42**	**0.11**
F21M12	NED	58	3	155–159	2	Clauss *et al.* (2002)	0.07	0.08	0.10	**0.09**	**0.25**
ICE10	VIC	58	3	119–121	2	Clauss *et al.* (2002)	0.01	0.01	0.03	0.31	**0.63**
ICE13	FAM	58	3	217–253	9	Clauss *et al.* (2002)	0.32	0.36	0.64	**0.10**	**0.44**
F19G10	VIC	58	3	179–181	2	Clauss *et al.* (2002)	0.01	0.01	0.01	−0.01	0.01
AthZEPG	PET	58	3	126–165	15	Clauss *et al.* (2002)	0.36	0.41	0.73	**0.11**	**0.44**
nga151	FAM	58	3	92–104	5	Clauss *et al.* (2002)	0.02	0.03	0.03	**0.33**	0.02
nga129	FAM	58	3	140–160	3	Clauss *et al.* (2002)	0.02	0.01	0.19	−0.14	**0.93**
Overall	---	---	---	---	104	---	0.15	0.19	0.32	**0.16**	**0.42**

The primer concentration was 10 μM. The multiplex PCR was performed in a volume of 10 μL, containing 0.5 μL DNA, 5 μL Multiplex PCR Master Mix (QIAGEN) and 1 pmol of each primer. The PCR proceeded as follows: 15 min at 95 °C; 30 cycles of 30 s at 94 °C, 90 s at 53 °C, 1 min at 72 °C for multiplex set 1, or 35 cycles of 30 s at 94 °C, 90 s at 58 °C and 1 min at 72 °C for multiplex sets 2 and 3; and a final extension step of 30 min at 60 °C. The PCR products were loaded on an ABI 3130xl Genetic Analyzer with GS 500 LIZ size standard (Applied Biosystems, Foster City, CA, USA). Genotypes were determined using Gene Mapper version 4.0 software (Applied Biosystems). The genotypic data are provided in **Supporting Information** as supplementary material.

### Data analysis

We initially described the basic statistics for the population genetics of each sampling site and locus. We used GenoDive software version 2.0 (Meirmans and van Tienderen 2004) to calculate observed heterozygosity (*H*_o_), within-population genetic diversity (*H*_s_, identical to the expected heterozygosity [*H*_e_], calculated using GenoDive), total genetic diversity (*H*_t_) and the inbreeding coefficient (*G*_is_); and the Fstat version 2.9.3.2 (Goudet 2002) to calculate allelic richness (AR; El Mousadik and Petit 1996). Deviations of *G*_is_ from the Hardy–Weinberg equilibrium were analysed with 9999 one-sided permutation tests using GenoDive. We also analysed the number of alleles, genetic diversities and genetic differentiation among populations (*H*_o_, *H*_s_, *H*_t_ and *G*_st_) for each locus by using GenoDive. Significances of the genetic differentiation, *G*_st_, were analysed for each locus based on the sum of square of the test statistic with 9999 permutations. Linkage disequilibrium among the 19 loci was also tested with 1000 Markov Chain Monte Carlo (MCMC) iterations with 100 batches, using GENEPOP software version 4.2 (Raymond and Rousset 1995). Because of the possibility of clonality, the number of identical genotypes was checked by the clone assignment implemented in GenoDive.

Secondly, we used a Bayesian clustering and population phylogeny to infer patterns of genetic differentiation. We estimated population gene pools and ancestry using Structure software version 2.3.4 (Pritchard *et al.* 2000) for all samples. The estimated number of ancestral populations (*K*) was set from 1 to 25 with a 100 000-step burn-in and 300 000 MCMC iterations. Allele frequencies were assumed to be correlated among populations and an admixture model was chosen. This process was repeated 20 times for each *K*. The probable *K* value was determined using the Δ*K* statistic (Evanno *et al.* 2005). The mean log-likelihood and the Δ*K* statistics were calculated using Structure Harvester software version 0.6.93 (Earl 2012). We repeated this structure analysis using the 30 populations from the Kinki area to further determine the genetic structure at the local scale. Additionally, we estimated the population structure using a phylogenetic network depicted by the SplitsTree4 version 4.13.1 (Huson and Bryant 2006). The network was constructed on the basis of Nei *et al.*'s (1983) genetic distance (*D*_a_; calculated using GenoDive).

Thirdly, we analysed the relationships of genetic differentiation and environmental dissimilarity or geographical distance. We selected *G′′*_st_ as a metric of genetic differentiation to adjust *H*_s_-dependent variation in *G*_st_ values (Meirmans and Hedrick 2011). The data from the entire study area and the Kinki area were separately analysed. According to the method of Rousset (1997), we converted pairwise *G′′*_st_ values into *G′′*_st_/(1 − *G′′*_st_) for each pair of sampling sites. The Mesh Climate 2000 database (Japan Meteorological Agency 2002) was used to compile the environmental variables. This database provides the information on air temperature (°C; daily mean, maximum and minimum), annual precipitation (mm), radiation (h), sunshine (MJ/m^2^) and maximum snow cover (cm) at a 1-km^2^ scale, and is thus suitable for our sampling scale. We added the altitude of each sampling site to these variables. These variables were summarized by a principal component analysis (PCA) to deal with multicollinearity of the environmental variables. The sum of the first and second principal components (PC1 and PC2) explained 78 and 79 % (respectively for the entire area and the Kinki area) of the total variation in habitat environments represented by the eight variables. PC1 explained ∼54 % of the variation, whereas PC2 explained roughly 25 % of the variation for both the entire area and the Kinki area. PC1 was related to the three temperature variables (daily mean, maximum and minimum air temperature) as well as the altitude, whereas PC2 was related to the light conditions (radiation and sunshine) and water availability (precipitation and snow cover). This tendency was consistent between the entire study area and the Kinki area (Fig. 1C and D). On the basis of the PCA results, we used PC1 and PC2 as representative indices to analyse genetic differentiation along environmental gradients. We used the Mantel tests to analyse Pearson's correlation between genetic and geographical distances; or partial Mantel tests for the correlation between genetic distance and the difference of principal component values by incorporating the geographical distance as a covariate. *P*-values were determined by 9999 permutations. We utilized the prcomp function for the PCA and the vegan package (Oksanen *et al*. 2013) implemented in the R software version 3.0.1 (R Core Team 2013) for the Mantel tests. Additionally, to deal with the exaggerated sampling on the Kinki area, we repeated the Mantel tests (with 999 permutations) for the entire study area by randomly selecting 10 populations from the Kinki area. This subsampling analysis was run 9999 times, where the significance of test statistics (*r* and *P*) was determined by mean and 95 % CI (i.e. 1.96 × SD) of the resampled distribution.

Finally, we estimated demographic changes in each studied population using the Msvar programme version 1.3 (Beaumont 1999). On the basis of a coalescent simulation by hierarchical Bayesian models with a step-wise mutation assumption, this programme provides multi-locus posterior distribution estimates for current and ancient effective population size (log_10_[*N*_0_]) and log_10_[*N*_1_]), respectively), mutation rate (log_10_[*μ*]) and the time since populations started to expand/decline (log_10_[*T*_a_]). These parameters were allowed to vary among all 19 loci, while their initial values were equal among these loci. The generation time (*g*_a_) was set as 2 years because *A. halleri* subsp. *gemmifera* is a perennial herb able to bloom during the first year of its life cycle under cultivated conditions (Y. Sato, pers. obs.). We used uninformative lognormal priors as follows: means of the prior distributions were set to 4, 4, −4 and 5; and SD equal to 4, 4, 1 and 4 for *N*_0_, *N*_1_, *μ* and *T*_a_, respectively. Hyper-prior lognormal distributions were set as: mean equal to 6, 6, −4 and 8; SD equal to 2, 2, 0.25 and 2; hyper-prior of mean equal to 0, 0, 0 and 0; hyper-prior of SD equal to 0.5, 0.5, 0.5 and 0.5 for *N*_0_, *N*_1_, *μ* and *T*_a_, respectively. Three independent chains of MCMC were run with 40 000 thinned updates and a thinning interval of 20 000 steps (i.e. total number of update steps = 8 × 10^8^). The coda package (Plummer *et al.* 2006) implemented in R was used to estimate the posterior distribution and to assess convergence among three chains, where the first half of the iterations was discarded as burn-in periods. The convergence was assessed using potential-scale reduction factors (Gelman and Rubin 1992). For the coalescent estimation, we did not analyse populations with less than seven individuals due to their small sample size (total number of populations analysed = 30).

## Results

### Basic statistics of population genetics

The AR ranged from 1.00 to 2.02 in all populations (Table 1). Within-population genetic diversities were <0.4 for all populations. *G*_is_ values significantly deviated from zero in 23 of 41 populations (Table 1). A few populations were found to have private alleles (Table 1). Percentages of null alleles were <5 %, except for 7.5 % in Site 19. Most markers showed significant genetic differentiation among the studied populations (global *G*_st_ = 0.42; Table 2). Sixteen markers showed a significant *G*_is_, and six of them had *G*_is_ values >0.2 (Table 2). No marker pairs exhibited a significant linkage disequilibrium, except in three cases (ICE12-MHJ24, *χ*^2^ = 14.4, d*f* = 6, *P* = 0.006; ICE10-AthZEPG, *χ*^2^ = 6.05, d*f* = 2, *P* = 0.048; ATH-AthCTR1A, *χ*^2^ = 91.9, d*f* = 34, *P*< 0.0001). Within the entire dataset, there were only five individuals with missing values at more than four loci. Although Site 40 consisted of identical genotypes across the 19 loci, identical genotypes were <10 % in the entire dataset.

### Patterns of population genetic structure

**Figure 2.**
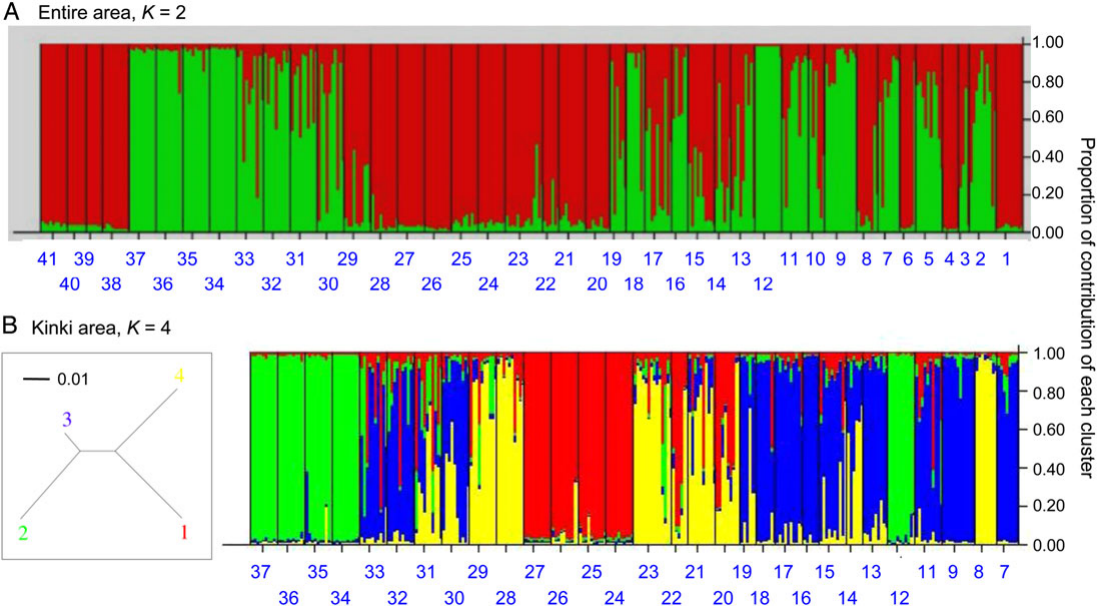
Genetic groups inferred from the structure analysis (Pritchard *et al.* 2000). (A) and (B) show results at the number of ancestral populations (*K*) = 2 and 4 for the entire study area and within the Kinki area, respectively. The inset of (B) presents a neighbour-joining tree based on allele-frequency divergence among clusters (the scale bar indicates a 0.01 unit). The site IDs correspond to those in Table 1 and Fig. 1. Note that colours of clusters do not correspond between different values of *K*.

We observed the largest value of Δ*K* at *K* = 2 for all the 41 populations and at *K* = 4 for the 30 populations from the Kinki area **[****see Supporting Information****]**. At *K* = 2, most populations near and outside the Kinki area were assigned to one of two clusters, but admixture patterns of these two clusters were observed near the Gifu and Fukui prefectures of the Kinki area (Fig. 2A). Within the Kinki area, the structure results at *K* = 4 showed some distinct clusters and admixed patterns. Four populations located in the Kyoto (Sites 24–27) and Hyogo prefectures (Sites 34–37) were clearly assigned to different groups (Clusters 1 and 2, respectively; an inset of Fig. 2B). While admixed patterns were observed in other sites, a single cluster (Cluster 4) was predominant near the boundary between the Kyoto and Shiga prefectures (Fig. 2B). Another cluster (Cluster 3) was predominantly observed in the eastern Shiga and Osaka areas (Fig. 2B). We also found two sites that were assigned to genetic groups different from their neighbouring sites within the Kinki area (Sites 8 and 12; Fig. 2B). The expected heterozygosity of individuals within the same cluster ranged from 0.19 to 0.31 within the Kinki area (Clusters 1–4 = 0.20, 0.25, 0.28 and 0.30, respectively). Genetic differentiation from an estimated ancestry ranged from 0.06 to 0.42 (*F*_st_ = 0.42, 0.42, 0.06 and 0.17 for Clusters 1–4, respectively).

**Figure 3.**
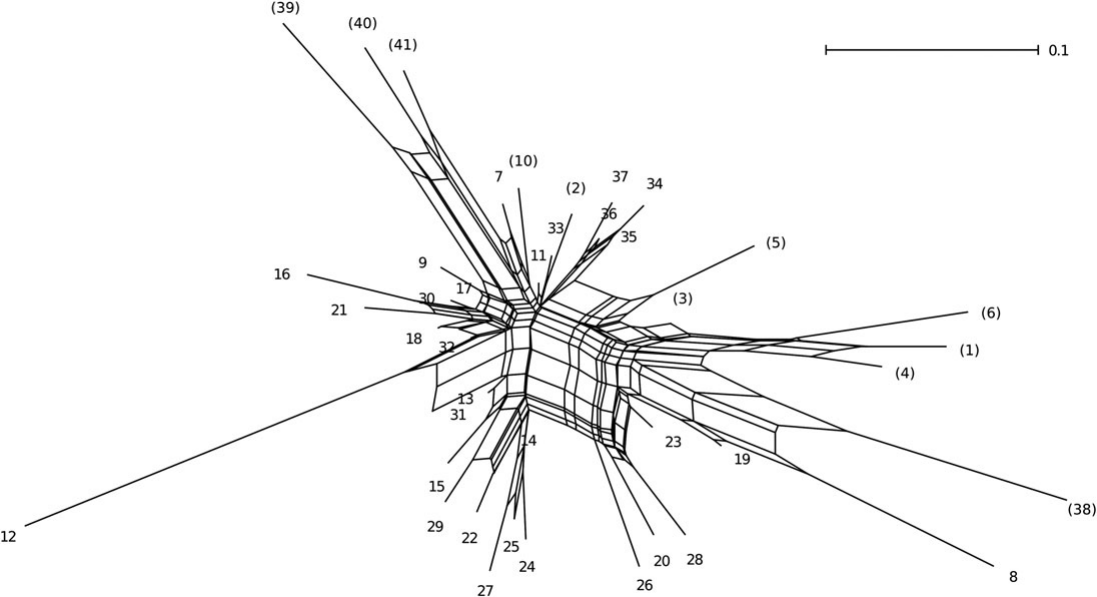
A split phylogenetic network between the studied *A. halleri* subsp. *gemmifera* populations based on Nei *et al.*'s (1983) *D*_a_ distances. The site IDs correspond to Table 1 and Fig. 1. The IDs of sites outside the Kinki area are within parentheses. The scale bar indicates a 0.1 unit of the *D*_a_ distance.

In the phylogenetic network (Fig. 3), we found multiple but not clear clusters within the Kinki area (Sites 7–9 and 11–37). The populations outside the Kinki area (Sites 1–5, 10 and 38–41) were located on margins of the network. The north-eastern populations (Sites 1 and 3–6) tended to cluster in the upper right of the network, while the south-western populations (Sites 39–41) were grouped in the upper left. Within the Kinki area, there were two populations that were genetically distant from the others (Sites 8 and 12; Fig. 3), corroborating the structure result that these two sites were assigned to genetic groups different from the neighbouring ones (*K* = 4 for the Kinki area; Fig. 2B).

### Isolation-by-distance or isolation-by-environment

**Figure 4.**
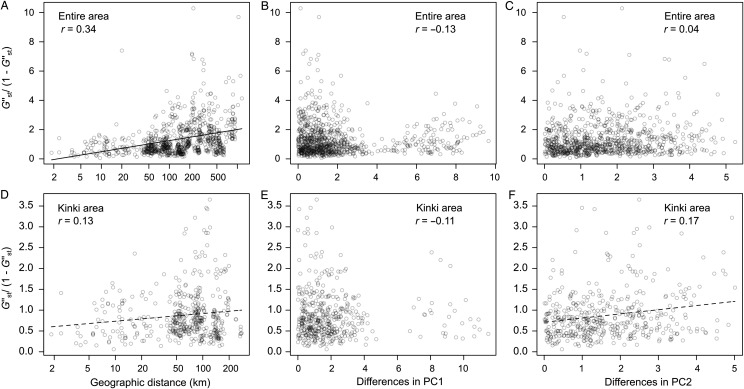
Isolation-by-distance or -environmental gradients across the Japanese mainland (A–C) and within the Kinki area (D–F). Pairwise genetic distances (*G′′*_st_/[1 − *G′′*_st_]) are plotted against geographical distances (km) or the differential values of the first or second principal components (PC1 and 2; see also Fig. 1C and D). Solid and dashed lines indicate significant (*P* < 0.05) and marginally significant trends (*P* < 0.1), respectively. Pearson's correlation coefficient (*r*) is also shown within each panel.

The degree of genetic differentiation showed a significantly positive correlation with the geographical distance (Mantel tests, *r* = 0.34, *P* < 0.001 with 9999 permutations; Fig. 4A), but was not correlated significantly with the environmental components among the entire study area (partial Mantel tests, *r* = –0.13, *P* = 0.88; *r* = 0.04, *P* = 0.30 for PC1 and PC2, respectively; Fig. 4B and C). These results were also supported when we subsampled the Kinki populations (geographical distance, *r* = 0.25 ± 0.06, *P* = 0.03 ± 0.04; PC1, *r* = –0.13 ± 0.07, *P* = 0.77 ± 0.16; PC2, *r* = –0.08 ± 0.14, *P* = 0.72 ± 0.37: Mean ± 95 % CI by 9999 times subsamplings). On the other hand, within the Kinki area, the genetic differentiation was marginally correlated with the geographical distance (Mantel tests, *r* = 0.13, *P* = 0.099; Fig. 4D). The genetic differentiation within the Kinki area was not correlated with the difference in the environmental PC1 value (partial Mantel tests, *r* = –0.11, *P* = 0.78; Fig. 4E) but positively correlated with the PC2 values at a marginally significant level (*r* = 0.17, *P* = 0.054; Fig. 4F). Although the high altitude populations (Sites 5–7) were outliers in the environmental PC1 (Fig. 1C and D), significant correlations were not detected in the PC1 difference even when the altitude variable was excluded from the PCA (*r* = –0.16, *P* = 0.93; *r* = –0.10, *P* = 0.74, for the entire area and Kinki samples, respectively).

### Demographic changes of local populations

The coalescence-based analysis by the Msvar programme indicated that most populations experienced a 10^2^ to 10^3^ times reduction in the effective population size compared with the ancestral (Table 1). The time since the demographic changes was estimated at 10^4^–10^5^ years, except for a few populations (>10^8^ years for Sites 34 and 41; Table 1). Because estimations for four populations did not converge (multivariate potential scale reduction factors >1.1 for Sites 2, 12, 31 and 31), further MCMCs were run with 120 000 thinned updates for these populations. After this additional run, the multivariate potential-scale reduction factors reduced to ≤1.1 for all populations, indicating successful convergence among the MCMC chains.

## Discussion

We did not detect more than two genetic groups across the Japanese mainland, but we identified a fine-scale differentiation and admixed pattern within the central area, as indicated by the structure analysis. The magnitude of genetic differentiation depended on geographical distance, but not on the environmental differences across the entire study scale. However, at least within the central area, we found suggestive evidence for environment-related differentiation, although it was marginally significant. Given that the second principal component was related to the light conditions (radiation and sunshine) and water availability (precipitation and snow cover), we hypothesize that microhabitat conditions can be a factor in local adaptation. Moreover, *A. halleri* subsp. *gemmifera* often occurs in transient habitats such as vegetation gaps (Ihara 1976), and we indeed observed them often growing in gravel along valleys or shady habitats nearby forest margins. Hence, it seems plausible that microhabitat environments rather than meteorological conditions (such as temperature or altitude) influenced the distribution of our studied species and accordingly resulted in the isolation-by-environments.

In plants, isolation-by-adaptation has been suggested for micro-environmental regimes, such as salt tolerance in *Mimulus guttatus* (Lowry *et al.* 2008) or water availability in *Boechera stricta* (Lee and Mitchell-Olds 2011, 2013). These examples may agree with our evidence in which microhabitat-related differentiation was observed at a local scale. Furthermore, in the relative *A. thaliana*, the influence of breeding systems on genetic structure has been suggested as relevant even within a scale <50 km (Bomblies *et al.* 2010). In our studied species, it is possible that the life-history traits can facilitate the formation of the fine-scale differentiation. Specifically, *A. halleri* subsp. *gemmifera* has a potential for clonal growth (Aikawa *et al.* 2010) and thereby can generate identical genotypes within a population. Despite the fact that such identical genotypes were rare in the entire dataset, similar genotypes easily occurred in our studied clonal species. In addition, seeds of *A. halleri* subsp. *gemmifera* are not specialized for long-distance dispersal. Thus, asexual breeding system may have allowed populations from a small number of founders with low genetic diversity to become established. In total, combinations of microhabitat isolations and negative distance-dispersal relationships may be specific factors facilitating the local-scale differentiation in *A. halleri* subsp. *gemmifera*.

The coalescence-based estimation inferred a decline in the effective population size for most sites. Accompanied with the species' life-history discussed above (clonality and lack of long-distance dispersal), such a decline of population size could account for significantly positive *G*_is_ and moderate heterozygosity as it might have caused genetic drifts. In Japan, bottlenecks that are associated with positive inbreeding were also reported for a clonal self-incompatible herb *Veratrum album* subsp. *oxysepalum* (Kikuchi *et al.* 2013). The bottleneck effects exerted by the reduction of effective population size may therefore explain the local-scale genetic differentiation and low levels of within-population genetic diversity in our studied clonal self-incompatible species, *A. halleri* subsp. *gemmifera*. However, other factors may also be responsible for the within- and among-population patterns of genetic diversity. For example, in a self-incompatible relative *A. lyrata*, selfing has recently evolved in some populations (Mable and Adam 2007; Foxe *et al.* 2010) and has consequently influenced their genetic structure (Willi and Määttänen 2011). Although the evidence for the breakdown of self-incompatibility has not been reported in our studied species yet, this factor remains possible for some populations exhibiting the extremely positive deviation of *G*_is_ (e.g. Sites 20 and 21).

The pattern of genetic structure of *A. halleri* subsp. *gemmifera* seems comparable with those of the temperate deciduous broadleaved trees in Japan, i.e. admixed pattern in central Japan (Hiraoka and Tomaru 2009*b*; Iwasaki *et al.* 2010). A comparative phylogeographic study on four species of deciduous broadleaved trees has identified genetic groups specific to the coastal area of the Kinki region; this postulates the presence of multiple refugia within the Kinki area during glacial periods (Iwasaki *et al.* 2012). The phylogeographical scenario for the temperate deciduous broadleaved trees may be responsible for the presence of multiple genetic groups that we identified within the Kinki area. Despite the unclear genetic structure across the Japanese mainland, significant isolation-by-distance was detected across the entire study area. This may suggest that the genetic differentiation have been formed gradually along the geographic scale rather than clustered into distinct genetic groups.

The present results should be interpreted with caution. Firstly, the sample size per site in several populations was too small to accurately quantify their genetic variation or perform quantitative analysis. Secondly, our structure analysis detected an admixture of multiple genetic groups even within a population (e.g. Sites 7, 23, 29 and 31). The underlying genetic structure made it difficult to accurately estimate the demographic processes of highly admixed populations. Thirdly, we should note that our demographic inference has some limitations due to a simplified assumption. In particular, the Msvar estimates assumed the absence of gene flow among local populations (Beaumont 1999). Thus, this assumption prevented us from excluding a possibility that the decline of population size and the time since the demographic change were overestimated for proximate populations in the Kinki area, while the estimates of regional samples did not differ considerably from those of local ones. These caveats should be taken into account when interpreting the patterns of genetic differentiation reported here.

## Conclusions

In summary, this study provides suggestive evidence for microhabitat-related differentiations at a local scale despite the lack of isolation-by-environment across the Japanese mainland in *A. halleri* subsp. *gemmifera*. We also suggest the possible effects of population decline on the patterns of genetic variation. Our findings therefore highlight a potential impact of ecological and demographic factors on the genetic differentiation at a fine spatial scale. Further investigations (such as common garden experiments) are required for rigorous testing of isolation-by-adaptation in order to understand how local adaptation and geographic isolation interactively affect the pattern of genetic differentiation within a plant species.

## Sources of Funding

This study was supported by funding Programme for Next Generation World-Leading Researchers (NEXT Program, GS013), JSPS and by Grant-in-Aid for Scientific Research (S) 26221106, MEXT to H.K.

## Contributions by the Authors

Y.S. performed the experiment and analysed the data. Y.S. and H.K. designed the study and wrote the manuscript.

## Conflicts of Interest Statement

None declared.

## Supporting Information

The following Supporting Information is available in the online version of this article –

**Figure S1.** Pictures of the study sites and plants.

**Figure S2.** Log-likelihood and Δ*K* values showing the likely number of ancestral populations inferred by the structure analysis (Pritchard *et al.* 2000).

**Figure S3.** Genepop format of raw genotypic data [SatoKudoh_AhSSR_GENEPOP.txt].

Additional Information

## References

[PLU070C1] Aikawa S, Kobayashi MJ, Satake A, Shimizu KK, Kudoh H (2010). Robust control of the seasonal expression of the *Arabidopsis FLC* gene in a fluctuating environment. Proceedings of the National Academy of Sciences of the USA.

[PLU070C2] Beaumont MA (1999). Detecting population expansion and decline using microsatellites. Genetics.

[PLU070C3] Bell CJ, Ecker JR (1994). Assignment of 30 microsatellite loci to the linkage map of *Arabidopsis*. Genomics.

[PLU070C4] Bomblies K, Yant L, Laitinen RA, Kim ST, Hollister JD, Warthmann N, Fitz J, Weigel D (2010). Local-scale patterns of genetic variability, outcrossing, and spatial structure in natural stands of *Arabidopsis thaliana*. PLoS Genetics.

[PLU070C5] Clauss MJ, Cobban H, Mitchell-Olds T (2002). Cross-species microsatellite markers for elucidating population genetic structure in *Arabidopsis* and *Arabis* (Brassicaeae). Molecular Ecology.

[PLU070C6] Dobson M (1994). Patterns of distribution in Japanese land mammals. Mammal Review.

[PLU070C7] Doyle JJ, Doyle JL (1987). DNA extraction by using DTAB-CTAB procedures. Phytochemical Bulletin.

[PLU070C8] Earl DA (2012). STRUCTURE HARVESTER: a website and program for visualizing STRUCTURE output and implementing the Evanno method. Conservation Genetics Resources.

[PLU070C9] El Mousadik A, Petit RJ (1996). High level of genetic differentiation for allelic richness among populations of the argan gree (*Argania spinoda* (L.) Skeels) endemic to Morocco. Theoretical and Applied Genetics.

[PLU070C10] Emanuelli F, Lorenzi S, Grzeskowiak L, Catalano V, Stefanini M, Troggio M, Myles S, Martinez-Zapater JM, Zyprian E, Moreiral FM, Grandol MS (2013). Genetic diversity and population structure assessed by SSR and SNP markers in a large germplasm collection of grape. BMC Plant Biology.

[PLU070C11] Evanno G, Regnaut S, Goudet J (2005). Detecting the number of clusters of individuals using the software STRUCTURE: a simulation study. Molecular Ecology.

[PLU070C12] Foxe JP, Stift M, Tedder A, Haudry A, Wright SI, Mable BK (2010). Reconstructing origins of loss of self-incompatibility and selfing in north American *Arabidopsis lyrata*: a population genetic context. Evolution.

[PLU070C13] Fujii N, Senni K (2006). Phylogeography of Japanese alpine plants: biogeographic importance of alpine region of Central Honshu in Japan. Taxon.

[PLU070C14] Garrido JL, Fenu G, Mattana E, Bacchetta G (2012). Spatial genetic structure of *Aquilegia* taxa endemic to the island of Sardinia. Annals of Botany.

[PLU070C15] Gelman A, Rubin DB (1992). Inference from iterative simulation using multiple sequences. Statistical Science.

[PLU070C16] Goto K, Shimamoto K, Okada K, Tabata T (2005). DNA and RNA extraction for *Arabidopsis thaliana*. Experimental protocols for model plants.

[PLU070C17] Goudet J (2002). http://www2.unil.ch/popgene/softwares/fstat.htm.

[PLU070C18] Hiraoka K, Tomaru N (2009a). Genetic divergence in nuclear genomes between populations of *Fagus crenata* along the Japan sea and Pacific sides of Japan. Journal of Plant Research.

[PLU070C19] Hiraoka K, Tomaru N (2009b). Population genetic structure of *Fagus japonica* revealed by nuclear microsatellite markers. International Journal of Plant Sciences.

[PLU070C20] Hewitt GM (2000). The genetic legacy of the quaternary ice ages. Nature.

[PLU070C21] Hoffmann MH (2005). Evolution of the realized climatic niche in the genus: *Arabidopsis* (Brassicaceae). Evolution.

[PLU070C22] Huson DH, Bryant D (2006). Application of phylogenetic networks in evolutionary studies. Molecular Biology and Evolution.

[PLU070C23] Ihara K (1976). Mode of local differentiation in *Arabis lyrata* and *A. gemmifera* (Cruciferae) in Japan. Journal of the Faculty of Science.

[PLU070C24] Iwasaki T, Tono A, Aoki K, Seo A, Murakami N (2010). Phylogeography of *Carpinus japonica* and *Carpinus tschonoskii* (Betulaceae) growing in Japanese deciduous broad-leaved forests, based on chloroplast DNA variation. Acta Phytotaxonomica et Geobotanica.

[PLU070C25] Iwasaki T, Aoki K, Seo A, Murakami N (2012). Comparative phylogeography of four component species of deciduous broad-leaved forests in Japan based on chloroplast DNA variation. Journal of Plant Research.

[PLU070C26] Japan Meteorological Agency (2002). Mesh Climate Data of Japan 2000.

[PLU070C27] Kikuchi R, Pak JH, Takahashi H, Maki M (2013). Pattern of population genetic structure revealed by nuclear simple sequence repeat markers in the understory perennial *Veratrum album* ssp. *oxysepalum* (Melanthiaceae) with a disjunct pattern of chloroplast DNA haplotypes. Biological Journal of the Linnean Society.

[PLU070C28] Kudoh H, Whigham D (1997). Microgeographic genetic structure and gene flow in *Hibiscus moscheutos* (Malvaceae) populations. American Journal of Botany.

[PLU070C29] Lee CR, Mitchell-Olds T (2011). Quantifying effects of environmental and geographical factors on patterns of genetic differentiation. Molecular Ecology.

[PLU070C30] Lee CR, Mitchell-Olds T (2013). Complex trait divergence contributes to environmental niche differentiation in ecological speciation of *Boechera stricta*. Molecular Ecology.

[PLU070C31] Llaurens V, Castric V, Austerlitz F, Vekemans X (2008). High paternal diversity in the self-incompatible herb *Arabidopsis halleri* despite clonal reproduction and spatially restricted pollen dispersal. Molecular Ecology.

[PLU070C32] Loridon K, Cournoyer B, Goubely C, Depeiges A, Picard G (1998). Length polymorphism and allele structure of trinucleotide microsatellites in natural accessions of *Arabidopsis thaliana*. Theoretical and Applied Genetics.

[PLU070C33] Lowry DB, Rockwood RC, Willis JH (2008). Ecological reproductive isolation of coast and inland races of *Mimulus guttatus*. Evolution.

[PLU070C34] Mable BK, Adam A (2007). Patterns of genetic diversity in outcrossing and selfing populations of *Arabidopsis lyrata*. Molecular Ecology.

[PLU070C35] Meeus S, Honnay O, Jaquemyn H (2012). Strong differences in genetic structure across disjunct, edge, and core populations of the distylous forest herb *Pulmonaria officinalis* (Boraginaceae). American Journal of Botany.

[PLU070C36] Meirmans PG, Hedrick PW (2011). Assessing population structure: *F*_st_ and related measures. Molecular Ecology Resources.

[PLU070C37] Meirmans PG, van Tienderen PH (2004). Genotype and Genodive: two programs for the analysis of genetic diversity of asexual organisms. Molecular Ecology Notes.

[PLU070C38] Nei M, Tajima F, Tateno Y (1983). Accuracy of estimated phylogenetic trees from molecular data. Journal of Molecular Evolution.

[PLU070C39] Nosil P, Daniel JF, Ortiz-Barrientos D (2009). Divergent selection and heterogeneous genomic divergence. Molecular Ecology.

[PLU070C40] Numata M (1974). The flora and vegetation of Japan.

[PLU070C41] Oksanen JF, Blanchet FG, Kindt R, Legendre P, Minchin PR, O'Hara RB, Simpson GL, Solymos P, Stevens MHH, Wagner H (2013). http://CRAN.R-project.org/package=vegan.

[PLU070C42] Platt A, Horton M, Huang YS, Li Y, Anastasio AE, Mulyati NW, Ågren J, Bossdorf O, Byers D, Donohue K, Dunning M, Holub EB, Hudson A, Corre VL, Loudet O, Roux F, Warthmann N, Weigel D, Rivero L, Scholl R, Nordborg M, Bergelson J, Borevitz JO (2010). The scale of population structure in *Arabidopsis thaliana*. PLoS Genetics.

[PLU070C43] Plummer M, Best N, Cowles K, Vines K (2006). CODA: convergence diagnosis and output analysis for MCMC. R News.

[PLU070C44] Pritchard JK, Stephens M, Donnelly P (2000). Inference of population structure using multilocus genotype data. Genetics.

[PLU070C45] R Core Team (2013). R: A language and environment for statistical computing.

[PLU070C46] Raymond M, Rousset F (1995). GENEPOP (version 1.2): population genetics for exact tests and ecumenicism. Journal of Heredity.

[PLU070C47] Rousset F (1997). Genetic differentiation and estimation of gene flow from F-statistics under isolation by distance. Genetics.

[PLU070C48] Sakaguchi S, Sakurai S, Yamasaki M, Isagi Y (2010). How did the exposed seafloor function in postglacial northward range expansion of *Kalopanax septemlobus*? Evidence from ecological niche modelling. Ecological Research.

[PLU070C49] Sakaguchi S, Takeuchi Y, Yamasaki M, Sakurai S, Isagi Y (2011). Lineage admixture during postglacial range expansion is responsible for the increased gene diversity of *Kalopanax septemlobus* in a recently colonised territory. Heredity.

[PLU070C50] Sharbel TF, Haubold B, Mitchell-Olds T (2000). Genetic isolation by distance in *Arabidopsis thaliana*: biogeography and postglacial colonization of Europe. Molecular Ecology.

[PLU070C51] Sobel JM, Chen GF, Watt LR, Schemske DW (2010). The biology of speciation. Evolution.

[PLU070C52] Symonds VV, Lloyd AM (2003). An analysis of microsatellite loci in *Arabidopsis thaliana*: mutational dynamics and application. Genetics.

[PLU070C53] Takahara H, Sugita S, Harrison SP, Miyoshi N, Morita Y, Uchiyama T (2000). Pollen-based reconstructions of Japanese biomes at 0, 6000 and 18,000 ^14^C yr BP. Journal of Biogeography.

[PLU070C54] Tsuchimatsu T, Kaiser P, Yew CL, Bachelier JB, Shimizu KK (2012). Recent loss of self-incompatibility by degradation of the male component in allotetraploid *Arabidopsis kamchatica*. PLoS Genetics.

[PLU070C55] Tsukada M, Huntley B, Webb T (1988). Japan. Vegetation history.

[PLU070C56] Vekemans X, Hardy OJ (2004). New insights from fine-scale spatial genetic structure analyses in plant populations. Molecular Ecology.

[PLU070C57] Willi Y, Määttänen K (2011). The relative importance of factors determining genetic drift: mating system, spatial genetic structure, habitat and census size in *Arabidopsis lyrata*. New Phytologist.

