# Supplementary material for: Fine-scale genetic differentiation of a temperate herb: relevance of local environments and demographic change
Source: AoB Plants. 2014 Nov 10;6:plu070. doi: 10.1093/aobpla/plu070 (PMC4262940; doi:10.1093/aobpla/plu070)
Supplement: Additional Information [file supp_6_plu070_index.html]

Fine-scale genetic differentiation of a temperate herb: relevance of local environments and demographic change — Additional Information 

# Fine-scale genetic differentiation of a temperate herb: relevance of local environments and demographic change

## Additional Information

Additional Information

**Files in this Data Supplement:**

- Genepop format of raw genotypic data  - txt file
- Additional Information - Docx file
- Supplementary Figure 1 - tif file
- Supplementary Figure 2 - png file
