# Supplementary material for: Fine-scale genetic differentiation of a temperate herb: relevance of local environments and demographic change
Source: AoB Plants. 2014 Nov 10;6:plu070. doi: 10.1093/aobpla/plu070 (PMC4262940; doi:10.1093/aobpla/plu070)
Supplement: Additional Information [file supp_plu070_plu070supp.docx]

**FIGURE LEGENDS FOR SUPPORTING INFORMATION**

**Figure S1.** Pictures of *Arabidopsis halleri* subsp. *gemmifera* and their natural habitats. (A and C) Forest margin along a road from Hanase to Ashu, Kyoto Prefecture: (B and D) Historical mine site at the Ikuno Ginzan (Ikuno Silver Mine), Hyogo Prefecture.

**Figure S2.** The probable number of ancestral populations inferred by the Structure analysis (Pritchard *et al.* 2000). Upper (A and B) and lower (C and D) panels present *∆K* (Evanno *et al.* 2005) and log-likelihood values, respectively, against the number of ancestral populations (*K*) for samples from the entire study area (A and C) and Kinki area (B and D). Vertical lines in (C) and (D) indicate standard deviation among twenty processes of MCMC iterations.
