# Supplementary figures and images for: Fine-scale genetic differentiation of a temperate herb: relevance of local environments and demographic change
Source: AoB Plants. 2014 Nov 10;6:plu070. doi: 10.1093/aobpla/plu070 (PMC4262940; doi:10.1093/aobpla/plu070)

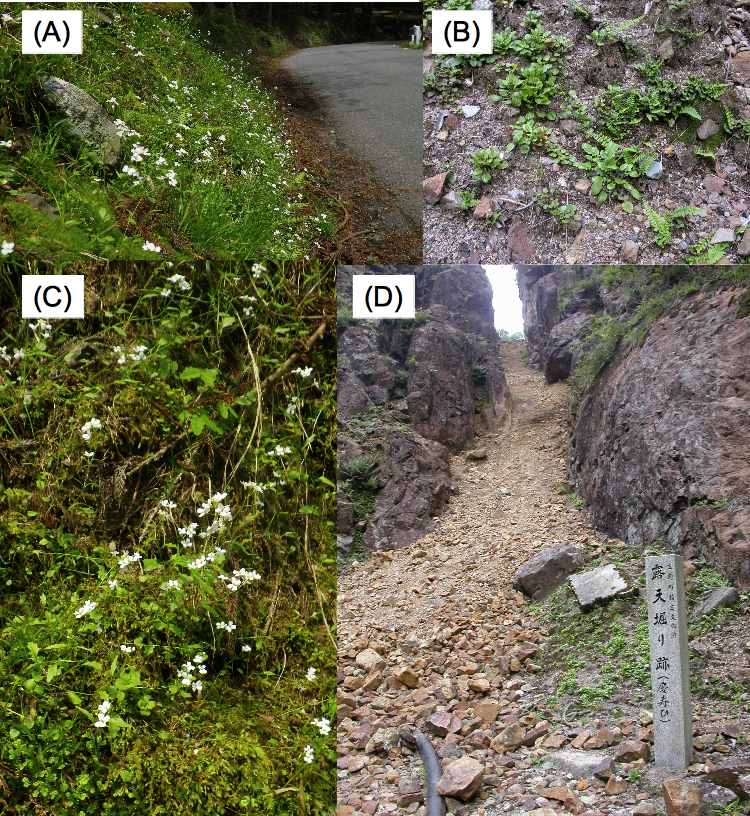

Supplement: Additional Information [file supp_plu070_plu070supp_fig1.tif]

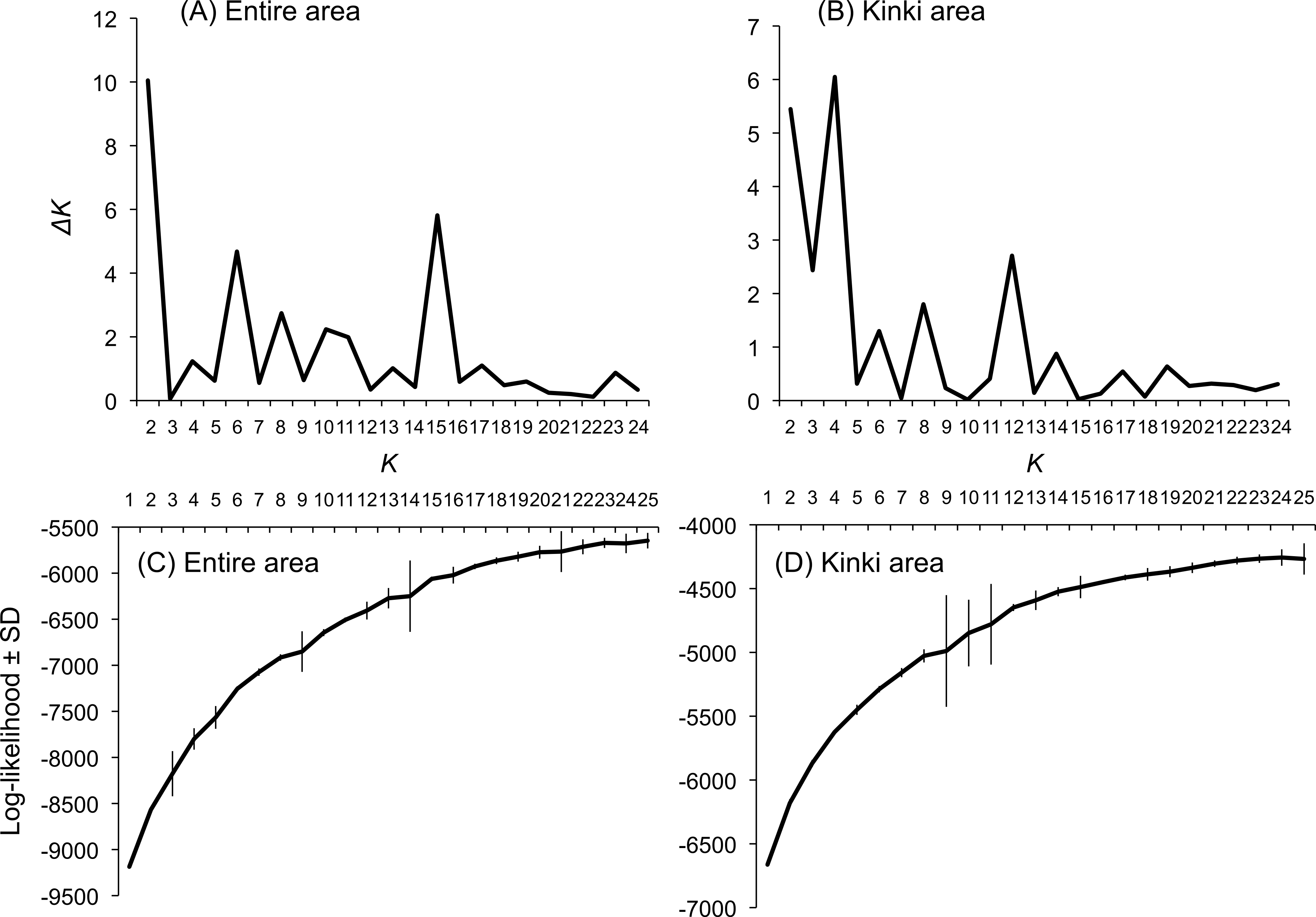

Supplement: Additional Information [file supp_plu070_plu070supp_fig2.png]
